# When a Safety Signal Appears: Do the Right Thing

**DOI:** 10.3389/fpubh.2013.00055

**Published:** 2013-11-13

**Authors:** John Franklyn Riefler

**Affiliations:** ^1^ICON Clinical Research, North Wales, PA, USA

**Keywords:** safety signal, antibiotics, antineoplastic, prolonged neuromuscular blockade, Vecuronium Bromide, twitch monitor

In this era of some pharmaceutical companies being accused of behaving poorly over the past two decades by hiding safety signals to maintain profits, it is refreshing to reflect back on a very different experience as an employe of Lederle Laboratories (now Pfizer). When in 1991 this company was informed by an investigator about a safety issue with piperacillin (Pipracil^®^), the response was adequate as I will demonstrate below.

I began my industry career in phase IV anti-infectives. We designed studies leading to new indications and tracked the safety of our products. I monitored all trials with three different antibiotics. One of my trials enrolled patients who had penetrating abdominal trauma (due to a knife wound or gunshot). We selected trauma surgeons as investigators who worked in large University trauma centers (“knife and gun clubs”). We expected cases with colonic penetration resulting in spillage of gut bacteria into the abdomen to benefit of piperacillin versus a comparator antibiotic.

Perioperatively, patients received one of the two study antibiotics plus vecuronium, a non-depolarizing neuromuscular blocking agent. An investigator informed me of the adverse event of prolonged neuromuscular blockade after an abdominal operation.

The investigator knew the Drug Interaction section in vecuronium’s package insert (1) included a statement that parenteral/intraperitoneal administration of high doses of certain antibiotics may intensify or produce neuromuscular blockade on their own. Penicillins, including piperacillin were not listed. If these or other newly introduced antibiotics are used in conjunction with vecuronium, unexpected prolongation of neuromuscular block should be considered a possibility. So, the investigator wanted to break the blind to determine if this subject received piperacillin.

My medical director asked a colleague uninvolved with this trial to break the blind. We discovered the patient received our drug. Vecuronium’s package insert (Adverse Reactions section) states the most frequent adverse reaction to non-depolarizing blocking agents as a class consists of an extension of the drug’s pharmacological action beyond the time period needed. This may vary from skeletal muscle weakness to profound and prolonged skeletal muscle paralysis resulting in respiration insufficiency or apnea. What should my company do with the knowledge of this adverse event reported from one of its clinical trials? Lederle, a very ethical big pharmaceutical company did the right thing. We designed and conducted a small study (30 subjects) to determine the magnitude of the neuromuscular blockade of piperacillin in combination with vecuronium. We included a positive control (an antibiotic known to cause this effect). We conducted the trial in a major university hospital. An anesthesiologist took twitch monitor measurements. This machine stimulates nerve causing muscle fibers to twitch; this allows a quantitative monitoring of the blockade.

The study confirmed piperacillin caused neuromuscular blockade and the control antibiotic showed the same amount of blockade as had been previously reported (2). We reported our findings to the FDA. This led to the following label change for piperacillin (3): When used in the perioperative period, piperacillin has been implicated in the prolongation of the neuromuscular blockade of vecuronium. Caution is indicated when piperacillin is used perioperatively. In one controlled clinical study, the ureidopenicillins, including piperacillin, were reported to prolong the action of vecuronium. Due to their similar mechanism of action, it is expected that the neuromuscular blockade produced by any of the non-depolarizing muscle relaxants could be prolonged in the presence of piperacillin.
